# Aberrant expression of NEK2 and its clinical significance in non-small cell lung cancer

**DOI:** 10.3892/ol.2014.2396

**Published:** 2014-07-30

**Authors:** XINWEN ZHONG, XIAOJIAO GUAN, WENKE LIU, LIN ZHANG

**Affiliations:** 1 Department of Thoracic Surgery, The First Clinical College, China Medical University, Shenyang, Liaoning, P.R. China; 2 Department of Pathology, The Second Clinical College, China Medical University, Shenyang, Liaoning, P.R. China; 3 Department of Pathology, Basic Science College, China Medical University, Shenyang, Liaoning, P.R. China

**Keywords:** NEK2, lung cancer, prognosis

## Abstract

The purpose of the present study was to identify a potential biomarker that is more effective than those already available for the prognosis of non-small cell lung cancer (NSCLC) patients. The expression of never in mitosis gene A (NIMA)-related kinase 2 (NEK2), minichromosome maintenance complex component 7 (Mcm7) and Ki67 was evaluated in 270 NSCLC tissues using immunohistochemical and immunofluorescence techniques. Associations between protein expression and clinicopathological characters were assessed, and the impact on overall survival was analyzed. High levels of NEK2, Mcm7 and Ki67 expression were detected in 25.9, 35.2 and 24.4% of the NSCLC tissues. Overexpression of NEK2 was detected more frequently in cases with high T and N stages (P<0.0001 and P=0.011, respectively). Correlations were present between the expression of NEK2, Mcm7 and Ki67. Kaplan-Meier curves indicated that the patients with overexpressed NEK2, Mcm7 and Ki67 had a poorer overall survival time compared to those with low expression for all stages (P<0.0001). In particular, the patients with NEK2 overexpression had a poorer prognosis. Multivariate Cox regression analysis showed that NEK2, Mcm7 and Ki67 are independent prognostic indicators for NSCLC. In conclusion, the data indicate that compared with Mcm7 and Ki67, NEK2 may be a more effective tumor proliferation marker of poor prognosis for NSCLC patients, and that NEK2 may represent a novel potential target for NSCLC therapeutic intervention.

## Introduction

Lung cancer is a highly lethal and extremely common cancer worldwide. A study of cancer statistics in 2011 reported that the overall 5-year survival rate of lung cancer patients was ~16% (1). Non-small cell lung cancer (NSCLC), of which adenocarcinoma and squamous cell carcinoma account for the vast majority of cases, represents almost 80% of primary lung cancer cases (2). Prediction of survival is mainly based on tumor stage. Even for patients diagnosed at stage I, the 5-year survival rate is <70% (3). It is critically important to identify robust, sensitive and specific biomarkers for prognosis in NSCLC. Identification of novel biomarkers may enhance early detection and effective treatment.

Never in mitosis gene A (NIMA)-related kinase 2 (NEK2) is a serine/threonine kinase located at the centrosome that functions by regulating centrosome cohesion and separation via the phosphorylation of its structural components. NEK2 exists in three forms, NEK2A, NEK2B and NEK2C, in mammalian cells (4). It is known that the aberrant regulation of NEK2 activity can lead to aneuploid defects and the abnormal proliferation of cancer cells (5). The majority of previous studies on NEK2 have been conducted in cell lines, but NEK2 has rarely been investigated in NSCLC (6).

Numerous studies have shown that cellular proliferative activity may provide valuable information for the prognosis and clinical management of various types of tumors, including NSCLC (7–12). Minichromosome maintenance complex component 7 (Mcm7) and Ki67 are two well-known cell proliferation markers. The former is expressed during the G_1_ to M phase of the cell cycle, and the latter appears in early G_1_ and persists in the S phase (13,14). In the present study, the expression levels of Mcm7, Ki67 and novel cell proliferation marker NEK2 were examined in NSCLC tissues, and the prognostic ability of these three proteins was investigated and compared.

## Materials and methods

### Clinical samples

A total of 270 patients who underwent a resection for NSCLC between 2006 and 2008 at the Department of Thoracic Surgery, The First Affiliated Hospital of China Medical University (Shenyang, Liaoning, China), were included in the present study. None of these patients received chemotherapy or radiotherapy prior to surgery. The group was composed of 192 males and 78 females, with a mean age of 62 years (range, 37–75 years) at the time of the surgery. A summary of the patient characteristics and the pathological characteristics is presented in Table I. Tumor specimens were either cut immediately after removal from the resected lung tissues, frozen in liquid nitrogen and then stored at −80°C, or collected in 10% formalin and embedded in paraffin for histopathological analysis. All 270 cases were independently classified as NSCLC by two experienced pathologists according to the World Health Organization histological typing criteria (15). The criteria for the tumor-node-metastasis (TNM) staging system was used to classify the clinicopathological factors and clinical stages of lung cancer (defined by the International Union Against Cancer TNM classification of malignant tumors, seventh edition, 2009) (15). All patients provided written informed consent and were subject to close follow-up observations. The median follow-up time subsequent to surgery was 60 months (range, 3 to 84 months). The study was approved by the Human Research Ethics Committee of China Medical University, which is accredited by the National Council on Ethics in Human Research.

### Immunohistochemistry and immunohistochemical assessment

Immunohistochemical studies on NEK2, Mcm7 and Ki67 were performed on formalin-fixed, paraffin-embedded tissue sections obtained from the aforementioned patients with NSCLC. Tissue sections were deparaffinized and then boiled in 0.01 mol/l sodium citrate buffer (pH 6.0) in a 1,000-watt microwave oven for 10 min to retrieve cell antigens. The primary antibodies used were rabbit polyclonal NEK2 antibody (1 to 200 dilution; Bioss, Beijing, China), mouse monoclonal Mcm7 antibody (1 to 200 dilution; Bioss) and mouse monoclonal Ki67 antibody (1 to 200 dilution; Maixin Biotechnology Development Co., Ltd., Fuzhou, China). The immunoco-expression of NEK2 with Mcm7 and Ki67 was analyzed using contiguous slices. All tissue sections were immunohistochemically stained using the avidin-biotin-peroxidase method and then counterstained with hematoxylin (Shenyang Shuangding Pharmaceutical Co., Ltd., Shenyang, China).

The staining was scored by three independent investigators without knowledge of patient outcomes. The sections were evaluated at low magnification (x100) to identify areas where NEK2, Mcm7 and Ki67 were evenly stained. The percentage of positively stained cells was calculated in >1, 000 tumor cells. The expression levels of Mcm7 and Ki67 were assessed by the labeling index, determined by counting the number of distinctly stained malignant cells, regardless of the intensity, divided by the total number of tumor cells. The two proteins were evaluated in the areas of highest positivity, and at least 1,000 tumor cells were counted. The average of the percentage of positive cells in the three scores represented the final score of the sample, yielding a continuous score from 0 to 100 for Mcm7 and Ki67. The expression of NEK2 was determined on the basis of staining intensity and the percentage of immunoreactive cells by reference to the immunoreactivity score (16). Staining intensity was rated as follows: 0, negative; 1, weakly positive; 2, moderately positive; and 3, strongly positive. The average tumor cell staining intensity score multiplied by the percentage of positive cells represented a final score ranging from 0 to 300. All cases were divided into two groups, a strongly-positive group (score range, 50–100 for Mcm7 and Ki67; and 240–300 for NEK2). All cases with discrepancies were jointly re-evaluated by the investigators and a consensus was obtained.

Assessment and imaging of the immunohistochemistry was performed using a Leica DM2000 microscope equipped with Leica DFC Cameras-Image Acquisition System (software V3.5.0; Leica Microsystems, Heerbrugg, Switzerland).

### Immunofluorescence

The sections were deparaffinized in xylene, rehydrated in graded alcohol series and boiled in 0.01 M citrate buffer (pH 6.0) for 2 min in an autoclave. Double immunofluorescence analysis was performed using rabbit polyclonal NEK2 antibody (1 to 200 dilution), mouse monoclonal Mcm7 antibody (1 to 200 dilution) and mouse monoclonal Ki67 antibody (1 to 200 dilution). Goat anti-rabbit (Alexa Fluor 488-labeled; Molecular Probes) and goat anti-mouse (Alexa Fluor 594-labeled; Molecular Probes, Invitrogen Life Technologies, Carlsbad, CA, USA) were used as the secondary antibodies. Fluorescence signals were analyzed by recording stained images using an Olympus FV1000 Laser Scanning Confocal Microscope (Olympus, Tokyo, Japan).

### Statistical analysis

The data were subject to statistical analysis using the SPSS software package (version 13.0; SPSS, Inc., Chicago, IL, USA). The correlation between the expression of NEK2 and Mcm7/Ki67 and the clinicopathological parameters was tested by χ^2^ test and bivariate analysis. Survival curves were calculated by the Kaplan-Meier product-limit estimate method and then examined using the log rank procedure. The significance of multiple predictors of survival was assessed by the Cox regression analysis. P<0.05 was considered to indicate a statistically significant difference.

## Results

### Expression of NEK2, Mcm7 and Ki67 in NSCLC, and clinicopathological features

**Figure 1:**
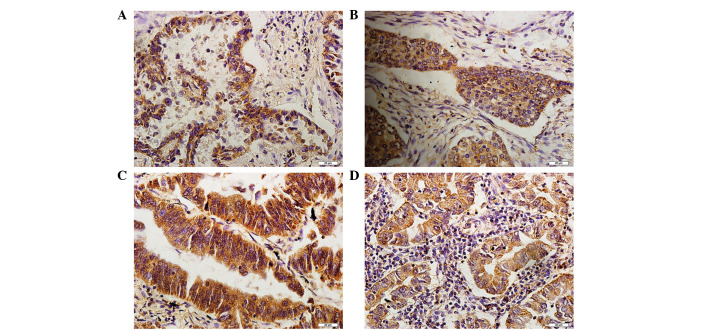
Immunohistochemical results of NEK2 in NSCLC. (A) The expression of NEK2 in moderately differentiated lung ADC. (B) The expression of NEK2 in moderately differentiated lung SCC. (C) The expression of NEK2 in bronchioalveolar carcinoma. (D) The expression of NEK2 in lung adenosquamous carcinoma. (bar, 20 μm). NEK2, never in mitosis gene A (NIMA)-related kinase 2; NSCLC, non-small cell lung cancer; SSC, squamous cell carcinoma; ADC, adenocarcinoma.

The immunohistochemistry staining for NEK2 was mostly positive in the cytoplasm of the tumor cells (Fig. 1). Meanwhile, positive immunohistochemical staining of Mcm7 and Ki67 was observed in the nucleus of the tumor cells. However, NEK2, Mcm7 or Ki67 were not expressed in normal bronchial epithelial cells. The correlation between the expression of NEK2, Mcm7, Ki67 and the clinicopathological characteristics of the patients with NSCLC is summarized in Table I. The results showed that the expression of the NEK2, Mcm7 and Ki67 proteins did not correlate with age, gender or histological grade. However, the expression of NEK2 was significantly correlated with the T stage and lymph node status (P<0.0001 and P=0.011, respectively).

### Correlation between the expression of NEK2, Mcm7 and Ki67

**Figure 2:**
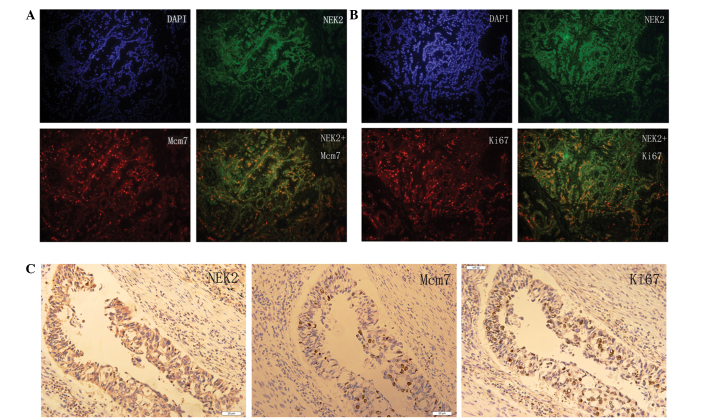
Immunoco-localization and co-expression images of NEK2 and Mcm7/Ki67 in NSCLC. (A) The co-localization of NEK2 and Mcm7 in well-differentiated lung ADC. (B) The co-localization of NEK2 and Ki67 in poorly-differentiated lung SCC. NEK2-positive staining was present in the cytoplasm and Mcm7/Ki67 in the nucleus. The coexistence regions were present in the majority of NEK2-positive NSCLC cells. (C) The co-expression of NEK2 and Mcm7/Ki67 in moderately-differentiated lung ADC (bar, 20 μm). NEK2, never in mitosis gene A (NIMA)-related kinase 2; mcm7, minichromosome maintenance complex component 7; NSCLC, non-small cell lung cancer; SSC, squamous cell carcinoma; ADC, adenocarcinoma.

NEK2 was located in the cytoplasm of the NSCLC cells and co-located with Mcm7 and Ki67, which were located in nucleus of the NSCLC cells. Fig. 2 presents the co-expression and co-localization of the expression of NEK2 and Mcm7/Ki67 in the NSCLC tissue. The correlation analysis between the expression of NEK2 and Mcm7/Ki67 in the NSCLC tissues is summarized in Table II. The results showed that positive NEK2 expression was significantly associated with positive Mcm7 and Ki67 expression (P<0.0001).

### Survival analysis and prognostic significance of the expression of NEK2, Mcm7 and Ki67

**Figure 3:**
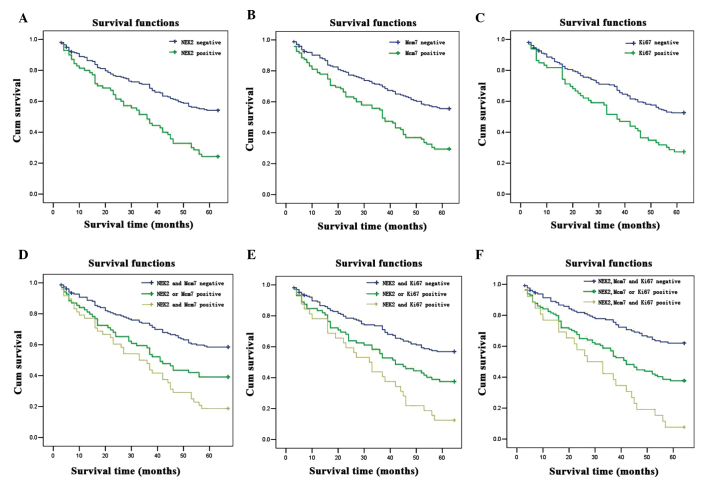
Kaplan-Meier curves of overall survival in NSCLC patients. (A) The 5-year overall survival rates were 55.0 and 24.3% in the patients with NSCLC with NEK2-negative expression (n=200) and NEK2-positive expression (n=70). (B) The 5-year overall survival rates were 56.6 and 29.5% in the patients with NSCLC with Mcm7-negative expression (n=175) and Mcm7-positive expression (n=95). (C) The 5-year overall survival rates were 53.4 and 27.3% in the patients with NSCLC with Ki67-negative expression (n=204) and Ki67-positive expression (n=66). (D) The 5-year overall survival rates were 59.5, 39.1 and 18.8% in the patients with NSCLC with NEK2- and Mcm7-negative expression (n=153), NEK2- or Mcm7-positive expression (n=69), and NEK2- and Mcm7-positive expression (n=48). (E) The 5-year overall survival rates were 57.8, 37.5 and 12.5% in the patients with NSCLC with NEK2- and Ki67-negative expression (n=166), NEK2- or Ki67-positive expression (n=72), and NEK2- and Mcm7-positive expression (n=32). (F) The 5-year overall survival rates were 63.1, 37.7 and 7.7% in the patients with NSCLC with NEK2-, Mcm7- and Ki67-negative expression (n=130), NEK2-, Mcm7- or Ki67-positive expression (n=114) and NEK2-, Mcm7- and Ki67-positive expression (n=26). There were significant differences between NEK2- and Mcm7/Ki67-positive, NEK2- or Mcm7/Ki67-positive, and NEK2- and Mcm7/Ki67-negative expression groups (P<0.0001).

The correlation between survival and the expression of NEK2, Mcm7 and Ki67 was evaluated in the 270 patients diagnosed with NSCLC. A significant difference was observed when the patient cohort was stratified by the level of the expression of NEK2, Mcm7 and Ki67. It was notable that the patients with NSCLC who had positive NEK2 and Mcm7/Ki67 expression had a lower survival rate than patients with NEK2- and Mcm7/Ki67-negative expression, indicating that NEK2 is a better prognostic factor than Mcm7/Ki67 (Fig. 3). Multivariate Cox regression analysis showed NEK2 expression was an independent prognostic factor for overall survival in patients with NSCLC (hazard ratio, 2.234; 95% confidence interval, 1.104–4.523; P=0.025), which was an improvement on the expression of Mcm7 (P=0.034) and Ki67 (P=0.026). However, the combined expression of NEK2 and Mcm7/Ki67 was an even more effective prognostic predictor (P<0.0001) (Table III).

## Discussion

During the past two decades, due to the histological and phonotypical heterogeneity of NSCLC, identification of more effective novel prognostic markers has become of vital importance in the selection of high-risk patients with NSCLC (15). Effective genetic markers can further stratify NSCLC into effective treatment subgroups. Prognosis may improve with a focus on the molecular markers of risk, which may lead to improved detection or treatment strategies (17,18).

It is known that the majority of tumor cells in human malignancies exhibit centrosome abnormalities. The deregulation of centrosome function may be a major contributory factor to cancer cell proliferation and progression (19). NEK2 is an important centrosome regulatory factor that was believed to be significant in finding a molecular mechanism for tumorigenesis and may now present a novel target for therapeutic intervention.

NEK2 is a serine/threonine kinase located at the centrosome and involved in mitotic regulation. NEK2 overexpression causes the induction of premature centrosome separation and nuclear defects, which are indicative of mitotic errors (20). NEK2 is involved in cell division and proliferation and mitotic regulation by centrosome splitting (21,22). Previous studies have also found that NEK2 protein expression is elevated 2–5 fold in cell lines derived from a variety of human tumors, including those of the ovary, breast and prostate (23). The present study showed that NEK2 expression was significantly upregulated in NSCLC. NEK2 expression was found to be correlated with T stage and lymph node metastasis. We have also found significant upregulated NEK2 expression in human breast cancer (unpublished data), which is indicated similarly in another previous study (24).

The Mcm7 protein is a type of licensing protein that can regulate DNA replication and indicates the presence of cell proliferation. The protein family includes 6 subunits, with Mcm7 as one of them; *in vivo* and *in vitro* experiments showed that once any member of the protein family becomes inactivated or missed, DNA replication will be repressed. They also have duplicate activity and play a crucial role in duplicate fork elongation (25–28). Abnormal Mcm7 expression is observed in numerous tumor types and correlates with a poor prognosis. Fujioka *et al* previously showed that Mcm7 in the tissues of lung adenocarcinomas could have prognostic implications (29). High MCM7 expression is also an adverse prognostic factor for overall survival in patients with Hodgkin lymphoma (HL) (30). Moreover, increasing MCM7 expression was observed from normal to cervical intraepithelial neoplasia III (CIN III) samples, with the highest MCM7 expression values detected in CIN III cases (31). Ki67 is a proliferation-associated nuclear antigen, whose expression can be observed in all cycling cells, with the exception of resting cells in the G_0_ phase, and expressed in cells in the S/G_2_ and M phase in particular. Ki67 has been widely identified as a parameter of tumor proliferation.

The present study demonstrated that NEK2, Mcm7, Ki67 and their combined expression appears to be associated with a poorer prognosis in patients with NSCLC. These proteins are independent prognostic factors for survival in patients with resected NSCLC, however, compared with Mcm7 and Ki67, NEK2 is a more effective proliferative factor for NSCLC prognosis. Moreover, a significant correlation was observed between the expression of the three proteins and the clinical pathological features. These results indicated that NEKs, Mcms and Ki67 may be involved in a relevant pathway in the tumor cell proliferation process, which requires further study. To the best of our knowledge, no study has previously been published concerning the association between NEK2 expression and patient prognosis in NSCLC cases; neither has a comparison been performed for the three cell proliferative proteins, NEK2, Mcm7 and Ki67.

In summary, the present study data revealed that NEK2, Mcm7 and Ki67 may all be independent prognostic factors in patients with NSCLC. NEK2 is a better factor for determining the prognosis of NSCLC. The evaluation of NEK2 expression may provide useful information for doctors to make optimal clinical decisions, and may be a novel potential target for NSCLC therapy, which will require analysis by further validation studies.

## Figures and Tables

**Table I:** Correlation between NEK2, Mcm7 and Ki67 expression and the clinicopathological features of NSCLC.

	Total		NEK2			Mcm7			Ki67		
				
Characteristics	n	%	Positive case	%	P-value	Positive case	%	P-value	Positive case	%	P-value
Age											
≤60	112	41.5	25	35.7	0.255	33	34.7	0.097	23	34.8	0.208
>60	158	58.5	45	64.3		62	65.3		43	65.2	
Gender											
Female	78	28.9	47	67.1	0.395	29	30.5	0.662	24	36.4	0.123
Male	192	71.1	23	32.9		66	69.5		42	63.6	
Histological type											
SCC	162	60.0	42	60.0	1.000	57	60.0	1.000	45	68.2	0.119
ADC	108	40.0	28	40.0		38	40.0		21	31.8	
Differentiation											
Well	59	21.9	14	20.0	0.277	23	24.2	0.729	16	24.2	0.600
Moderate	86	31.9	18	25.7		28	29.5		23	34.8	
Poor	125	46.3	38	54.3		44	46.3		27	40.9	
Tumor size											
T1	78	28.9	10	14.3	0.000^a^	19	20.0	0.017^a^	19	28.8	0.156
T2	143	53.0	37	52.9		52	54.7		30	45.5	
T3–4	49	18.1	23	32.9		24	25.3		17	25.8	
Lymph node metastasis											
Negative	143	53.0	27	38.6	0.011^a^	42	44.2	0.057	31	47.0	0.053
N1-positive	72	26.7	27	38.6		33	34.7		25	37.9	
N2–3-positive	55	20.4	16	22.9		20	21.1		10	15.2	
Metastasis											
M0	265	98.1	68	97.1	0.469	91	95.8	0.034^a^	63	95.5	0.062
M1	5	1.9	2	2.9		4	4.2		3	4.5	

a Significantly different.

NEK2, never in mitosis gene A (NIMA)-related kinase 2; mcm7, minichromosome maintenance complex component 7; NSCLC, non-small cell lung cancer; SSC, squamous cell carcinoma; ADC, adenocarcinoma.

**Table II:** Association between NEK2 and Mcm7/Ki67 expression in NSCLC.

	Mcm7				Ki67			
		
Characteristics	Positive case	Negative case	κ-value	P-value	Positive case	Negative case	κ-value	P-value
NEK2-positive case	48	22	46.188	<0.0001^a^	32	38	23.148	<0.0001^a^
NEK2-negative case	47	153			34	166		

a Significantly different.

NEK2, never in mitosis gene A (NIMA)-related kinase 2; mcm7, minichromosome maintenance complex component 7; NSCLC, non-small cell lung cancer.

**Table III:** Univariate and multivariate analysis of survival in 270 patients with NSCLC.

	Univariate analysis (n=270)		Multivariate analysis (n=270)	
		
Variable	Hazard ratio (95% CI)	P-value	Hazard ratio (95% CI)	P-value
NEK2-positive expression alone				
Negative vs. positive	3.810 (2.064–7.036)	<0.0001^a^	2.234 (1.104–4.523)	0.0250^a^
Mcm7-positive expression alone				
Negative vs. positive	3.117 (1.830–5.310)	<0.0001^a^	1.920 (1.050–3.512)	0.0340^a^
Ki67-positive expression alone				
Negative vs. positive	3.060 (1.667–5.617)	<0.0001^a^	2.179 (1.096–4.333)	0.0260^a^
NEK2- and Mcm7-positive expression				
NEK2- and Mcm7-negative vs. NEK2	6.360 (2.877–14.062)	<0.0001^a^	5.218 (2.264–12.026)	<0.0001^a^
Mcm7-positive vs. NEK2- and Mcm7-positive	2.786 (1.166–6.658)		2.402 (0.965–5.977)	
NEK2- and Ki67-positive expression				
NEK2- and Ki67-negative vs. NEK2	9.600 (3.221–28.610)	<0.0001^a^	7.836 (2.494–24.625)	<0.0001^a^
Ki67-positive vs. NEK2- and Ki67-positive	4.200 (1.328–13.280)		3.401 (1.026–11.276)	
Age				
<60 vs. ≥60	1.225 (0.754–1.991)	0.4120	1.136 (0.662–1.948)	0.6430
Gender				
Female vs. male	1.023 (0.604–1.733)	0.9330	1.076 (0.596–1.943)	0.8070
Histological type				
SCC vs. ADC	0.742 (0.454–1.211)	0.2330	0.663 (0.382–1.154)	0.1460
Tumor differentiation				
Poor vs. well or moderate	1.211 (0.750–1.957)	0.4340	1.359 (0.793–2.328)	0.2640
T stage				
I or II vs. III or IV	3.345 (1.657–6.754)	0.0010^a^	2.228 (1.020–4.867)	0.0440^a^
Lymph node status				
Negative vs. N1-positive vs. N2-positive	2.236 (1.370–3.649)	0.0010^a^	1.781 (1.038–3.054)	0.0360^a^
M stage				
M0 vs. M1	10×10^9^ (0.000)	0.9990	5×10^8^ (0.000)	0.9990

a Significantly different.

NEK2, never in mitosis gene A (NIMA)-related kinase 2; mcm7, minichromosome maintenance complex component 7; NSCLC, non-small cell lung cancer; SSC, squamous cell carcinoma; ADC, adenocarcinoma; CI, confidence interval.
